# Venoarterial extracorporeal life support in post-traumatic shock and cardiac arrest: lessons learned

**DOI:** 10.1186/1757-7241-22-12

**Published:** 2014-02-07

**Authors:** Yuan-His Tseng, Tzu-I Wu, Yuan-Chang Liu, Pyng-Jing Lin, Meng-Yu Wu

**Affiliations:** 1 Department of Cardiovascular Surgery, Chang Gung Memorial Hospital and Chang Gung University, 5, Fushing Street, Kueishan Hsiang, Taoyuan, Taiwan; 2 Department of Obstetrics and Gynecology, Wan Fang Hospital and Taipei Medical University, Taipei, Taiwan; 3 Department of Medicine, Taipei Medical University, Taipei, Taiwan; 4 Department of Medical Imaging and Intervention, Chang Gung Memorial Hospital and Chang Gung University, Taoyuan, Taiwan

**Keywords:** Extracorporeal life support, Extracorporeal membrane oxygenation, Cardiac arrest, Traumatic shock, Blunt chest trauma

## Abstract

**Objectives:**

Venoarterial extracorporeal life support (VA-ECLS) is an effective support of acute hemodynamic collapse caused by miscellaneous diseases. However, using VA-ECLS for post-traumatic shock is controversial and may induce a disastrous hemorrhage. To investigate the feasibility of using VA-ECLS to treat post-traumatic shock or cardiac arrest (CA), a single-center experience of VA-ECLS in traumatology was reported.

**Materials and methods:**

This retrospective study included nine patients [median age: 37 years, interquartile range (IQR): 26.5-46] with post-traumatic shock/CA who were treated with VA-ECLS in a single institution between November 2003 and October 2012. The causes of trauma were high-voltage electrocution (n = 1), penetrating chest trauma (n = 1), and blunt chest or poly-trauma (n = 7). Medians of the injury severity score and the maximal chest abbreviated injury scale were 34 (IQR: 15.5-41) and 4 (IQR: 3-4), respectively. All patients received peripheral VA-ECLS without heparin infusion for at least 24 hours.

**Results:**

The median time from arrival at our emergency department (ED) to VA-ECLS was 6 h (IQR: 4-47.5). The median duration of VA-ECLS was 91 h (IQR: 43-187) with a duration < 24 h in 2 patients. Among the 9 patients, 5 received VA-ECLS to treat the post-traumatic shock/CA presenting during (n = 2) or following (n = 3) damage-control surgeries for initial trauma, and another 4 patients were supported for non-surgical complications associated with initial trauma. VA-ECLS was terminated in 2 non-survivors owing to uncontrolled hemothorax or retroperitoneal hemorrhage. Three patients survived to hospital discharge. All of them received damage-control surgeries for initial trauma and experienced a complicated hospitalization after weaning off VA-ECLS.

**Conclusion:**

Using VA-ECLS to treat post-traumatic shock/CA is challenging and requires multidisciplinary expertise.

## Introduction

Extracorporeal life support (ECLS) is an effective treatment of acute but reversible cardiopulmonary failure caused by miscellaneous diseases [1-3]. This device simply sucks in deoxygenated venous blood and pumps oxygenated blood back into the patient’s circulation. The human coagulation system was strongly activated by ECLS owing to the blood-surface interaction, and systemic heparinization is necessary to reduce the burden of thromboembolism in the circulation [4]. To reduce the dosage of heparin administered intravenously, the heparin-bond ECLS circuit is adopted. Both the intravenous heparin and the heparin-bond circuit activate antithrombin III in the blood and induce a chain reaction of anti-coagulation [4]. ECLS offers two operating modes, depending on the destinations of the oxygenated blood, the venoarterial (VA) and the venovenous (VV) mode, for cardiopulmonary or purely pulmonary supports. In adult VA-ECLS, the oxygenated blood is pumped to the iliac artery (in most circumstances) to increase the arterial pressure and improve systemic perfusion. In VV-ECLS, the oxygenated blood is pumped to the right atrium and pulmonary circulation. This action provides a pre-pulmonary blood oxygenation and allows physicians to adopt “lung protective ventilation” in the face of acute lung injury (ALI). Recently, VV-ECLS has become accepted as an advanced support for post-traumatic ALI [5], but the role of VA-ECLS in traumatology has not been determined yet. Theoretically, VA-ECLS is harmful to patients in an uncontrolled bleeding shock because it may augment the hemorrhaging by transiently increasing the arterial pressure and worsen the early-type (fibrinolytic) traumatic coagulopathy [6] with its heparin-bond circuit. Two recent studies of using ECLS to treated patients with traumatic bleeding shock/CA show acceptable survivals, 14% (2/14) [7] and 66% (2/3) [8], in the VA group. However, none of the two studies provides clear information about the timing and strategies to activate this challenging resuscitation. To reduce this knowledge gap, a 9-year experience of using VA-ECLS in post-traumatic resuscitation was reviewed.

## Materials and methods

### Study population

From November 2003 to October 2012, 507 patients received ECLS in Chang-Gung Memorial Hospital and 27 of them received ECLS for post- traumatic hemodynamic or pulmonary support. Nine patients (median age: 37 years, interquartile rang [IQR]: 27-46) receiving VA-ECLS due to a refractory post-traumatic shock (systolic arterial pressure < 60 mmHg)/CA were enrolled in this retrospective study. The institutional review board of our hospital approved the study protocol (CGMF IRB No. 102-2919B).

### Exclusion criteria and management of VA-ECLS in trauma patients

Following the principle of “do no harm”, delivering VA-ECLS to trauma patients was contraindicated under the following conditions: (1) an out-hospital CA (OHCA) unresponsive to conventional resuscitations [9] and requiring a continuous cardiopulmonary resuscitation (CPR) > 30 minutes after arrival at our ED, (2) a bleeding shock/CA without adequate (at least temporary) control of the bleeders, or (3) an obvious intracranial hemorrhage (ICH). To rule out ICHs and occult internal bleedings, especially retroperitoneal hemorrhage, a whole body computed tomography (CT) was performed before delivering VA-ECLS to trauma patients with a possibility of bleeding shock.

Our practice of VA-ECLS has been described in detail previously [1,10]. Our ECLS team was composed of the duty cardiosurgeon and perfusionist. The ECLS device used was the Capiox emergent bypass system (EBS, Terumo Inc., Tokyo, Japan) with heparin-coated inner surface. The ECLS circuitry was de-aired with heparinized normal saline (2500 unit/1 L) before implantation. Patients with a low risk of bleeding would accept a loading dose of heparin (5000 unit) just before cannulation. A femoral -femoral VA cannulation was performed with two vascular cannula (DLP Medtronic, Minneapoli; inflow: 19-21 French, outflow: 17-19 French) using either a percutaneous Seldinger technique or direct cut-down method. The cut-down method was preferred in most cases because it provided a better vascular identification and bleeding control at the cannulation sites at the spastic femoral vessels. A small (6 French) distal perfusion catheter was also implanted to augment the distal arterial perfusion in the limb with arterial cannulation. Principally, the VA-ECLS was kept at maximal blood flow to achieve a mean arterial pressure between 80 ~ 60 mmHg initially. The oxygenator used pure oxygen as the sweep gas and the gas flow rate was titrated according to the results of arterial blood gas samplings to avoid respiratory alkalosis. To reduce the risk of bleeding during ECLS, the blood cell counts were checked daily to achieve a platelet count ≥ 80 k, hemoglobin ≥ 10 gm/dl, and fibrinogen ≥ 100 mg/dl. The active clotting time (ACT) and activated partial thromboplastin time (aPTT) were also tested periodically and systemic heparinization (the maintaining dose, from 500 u/h) would be preceded if the value of aPTT < 40 seconds. In patients with a low risk of bleeding, the ACT and aPTT were kept in the range of 160-180 and 40-55 seconds on VA-ECLS. In patients with a high risk of bleeding, a “heparin-minimized” strategy (no loading or maintaining dose of heparin) would be adopted. The length of the “heparin-free” strategy was not exceeded 48 h in our practice and a high-flow ECLS (blood flow > 2.5 L/min) must be maintained to reduce the risk of thrombosis. In-line continuous arteriovenous hemofiltration (CAVH) was offered to patients with oliguric/auric renal failure to achieve a modestly negative fluid balance. Weaning off VA-ECLS was attempted once myocardial contractility and systemic perfusion were improved. Patients showing a tolerance to a VA-ECLS flow of 1.5 L/min for 12 h were weaned from VA-ECLS. Termination of VA-ECLS would be undertaken in patients with profound brain damages, persistent hypotension with metabolic acidosis, or uncontrolled hemorrhages after acquiring the family’s consent. The VA-ECLS would be switched to VV-ECLS in selected patients showing adequate myocardial recovery but profound hypoxemia [arterial oxygen tension (P_a_O_2_)/fraction of inspired oxygen (F_i_O_2_) provided by mechanical ventilator ≤100 mmHg] during the process of weaning off VA-ECLS. The right internal jugular vein was cannulated with a 17 or 19 French cannula, and then the femoral artery was decannulated and repaired.

## Results

Table 1 summarizes the characteristics of trauma, indications of VA-ECLS, damage control interventions before/on VA-ECLS, and outcomes of the 9 patients. These patients had a median age of 37 years [interquartile range (IQR): 26.5-46] and miscellaneous trauma mechanisms. The median time from arrival at our emergency department (ED) to VA-ECLS was 6 h (IQR: 4-47.5). All patients but two had a whole body CT scan prior to ECLS to evaluate the severity of regional injuries. High-grade thoracic injury (median chest AIS: 4; IQR: 3-4) was common in our victims and often contributed to a high injury severity score (ISS; median ISS: 34; IQR: 15.5 - 41). One patient (case 1) with OHCA showed a fluctuating response to initial resuscitations at our ED and thus was given the highest ISS of 75. As shown in Table 1, all patients but one (case 4) had a CA unresponsive to conventional resuscitations and accepted VA-ECLS as the last resort. Five patients received damage-control surgeries for hemostasis before VA-ECLS. The median duration of VA-ECLS was 91 h (IQR: 43-187) with a duration < 24 h in 2 patients (cases 4 and 5). VA-ECLS was terminated in 2 patients owing to an uncontrolled retroperitoneal hemorrhage (case 4) or hemothorax (case 8). Three patients (33%) survived to hospital discharge. All of them received damage-control surgeries for initial trauma and experienced a complicated hospitalization after weaning off VA-ECLS.

**Table 1:** The characteristics of trauma, indication of venoarterial extracorporeal life support, and outcomes

**No.**	**Age/sex**	**ISS**	**Pre-VA-ECLS summary of major injuries**	**Pre-ECLS**	**Indication of VA-ECLS**	**Operation on VA- ECLS**	**Outcomes**
	**Injury mechanism**			**Damage-control surgery**			
	Time to VA-ECLS*						
1	21/M	75	OHCA (traumatic asphyxia)	No	CA in ED	No	Died-on-ECLS **(ECLS h:75)**
	Car accident		Bilateral lung contusion and HPnTx				
	3 h		Minor pelvic fracture				
2	37/M	10	Scalp lacerations	Right thoracotomy:	CA in OR	Re-thoracotomy for hemostasis	Survived **(ECLS h:112)**
	Chest stabbing		Right massive HTx	RLL wedge resection			
	4 h						
3	48/M	13	Right massive HTx,	Right thoracotomy	CA in OR	Cardiorrhaphy	Survived **(ECLS h:91)**
	Car accident		Grade 2 liver injury				
	4 h						
4	38/M	34	Coma (< 6 h),	No	Refractory hypothermia (31˚C)	No	Died-on-ECLS **(Termination of ECLS after 7 h)**
	Accidental fall		Bilateral lung contusion				
	5 h		Refractory hypothermia 31 ~ 30˚C				
5	29/M	41	Coma (≤ 6 h)	No	CA in ED	No	Died-on-ECLS **(ECLS h:17)**
	High-voltage electrocution		3^rd^ degree burn (35% TBSA)				
	6 h		Right open PnTx, bilateral lung contusion				
			Right femoral fracture				
6	33/M	36	Coma (> 6 h), Minor C-spine fracture,	Bilateral thoracotomy:	CA in ICU	Change to VV mode	Died-on-ECLS **(ECLS h:297)**
	Accidental fall		Bilateral lung contusion, ribs fractures, and HPnTx	RLL and LLL lobectomies			
	20 h		Minor pelvic fracture.				
7	47/F	41	Traumatic SAH	Right thoracotomy:	CA in OR	Reapir colonic perforation and end-ileostomy.	Died-on-ECLS **(ECLS h:69)**
	Motorbike accident		Bilateral lung contusion and HPnTx, Diaphragmatic rupture, grade 3 liver injury, grade 4 spleen injury, pelvic fracture with retroperitoneal hemorrhage, right radial fracture	Repair of RLL and diaphragmatic laceration			
	47 h			Laparotomy:			
				Splenectomy, hepatorrhaphy, retroperitoneal packing			
8	45/M	20	Bilateral lung contusion, flial chest, right HPnTx	No	CA in ICU	Change to VV mode	Died-on-ECLS **(Termination of ECLS after 258 h)**
	Motorbike accident		Right clavicle, ribs, left tibial and humeral fracture.				
	48 h						
9	24/M	18	Right lung contusion	Laparotomy:	CA in ICU (sepsis)	No	Survived **(ECLS h:116)**
	Motorbike accident		Mesenteric injury with small bowel gangrene	Segmental bowel resection and end-ileostomy			
	315 h		Minor L- spine fracture				

Time to VA-ECLS*: Emergency department admission to VA-ECLS deployment.

**ISS:** Injury severity score. **VA-ECLS:** venoarterial extracorporeal life support. **OHCA:** Out hospital cardiac arrest. **CA:** Cardiac arrest. **HTx:** Hemothorax. **PnTx:** Pneumothorax. **HPnTx:** Hemopneumothorax. **C-Spine:** Cervical spine. **L-spine:** Lumbar spine. **RML:** Right middle lobe. **RLL:** Right lower lobe. **LLL:** Left lower lobe.

**SAH:** Subarachnoid hemorrhage.

## Discussion

This study aims to investigate the feasibility of using VA-ECLS to treat post-traumatic shock/CA. VA-ECLS here is a desperate attempt to save patients from irreversibility and the decision to activate it is often made under time pressure [7,8]. Owing to the wide variety of presenting injuries and their corresponding managements, establishing an integrated protocol of VA-ECLS in traumatology can only be learned by exploration. According to our experience, post-traumatic shock/CA can result from (1) exsanguinations, (2) hypoxia, and (3) superimposed infections. Recognition of a pre-existing internal hemorrhage is essential while using VA-ECLS to treat post-traumatic shock/CA because the damage-control interventions should never be delayed in patients with traumatic bleeding shock [9]. The whole body CT scan is the preferred diagnostic tool here because it provides a noninvasive and expeditious screening of internal hemorrhages, especially the intracranial and retroperitoneal hemorrhages. A secure implantation of VA-ECLS is also necessary to prevent blood loss via the cannulation sites. Cannulation sites hemorrhage related to mal-cannulation is a possible explanation of the high failure rate (29%, 4/14) of VA-ECLS in a practice adopting percutaneous Seldinger technique exclusively [7], as seen in our case 4. The man was found unconscious without obvious injuries in the woods. The traumatologist requested VA-ECLS to correct the refractory hypothermia and hypotension after he excluded the possibility of significant intra-thoracic or intra-abdominal hemorrhages with chest radiography, sonography, and diagnostic peritoneal lavage. Unfortunately, the patient developed progressive abdominal distension with a decreasing ECLS flow after a percutaneous VA-ECLS. Either a vascular injury caused by mal-cannulation or an augmented hemorrhage from an undetected retroperitoneal hemorrhage should be responsible for his mortality. To avoid repeating this mistake, we now include a whole body CT scan prior to ECLS and the cut-down implantation as part of our standard practice for using VA-ECLS in traumatology.

**Figure 1:**
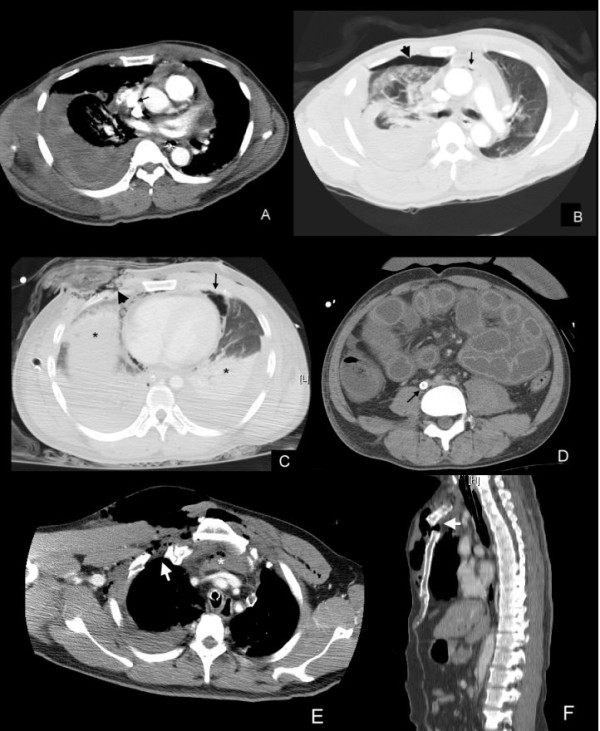
**Special computed tomographic images.** Blunt chest trauma: Hemothorax from a lacerated right atrium accompanied with a ruptured pericardium (Case 3). **A**. A massive right-sided hemothorax accompanied with a hemopericardium. The right auricle is surrounded by thrombus without extravasations of the contrast media into the right hemithorax (arrow). **B**. The coexistence of a right-sided pneumothorax (arrowhead) and a pneumopericardium (arrow) indicates a right-sided pericardial disruption in this case. Injuries in a victim of high-voltage electrocution (Case 5): **C**. An open pneumothorax with a defect of the right chest wall (arrowhead). Pulmonary consolidations (*) and pneumomediastinum (arrow) are also presented. **D**. The “shock bowel” appearance. An ECLS cannula is in the inferior vena cava (arrow). Blunt chest trauma: A flail chest from injuries of the sternoclavicular articulation (Case 8). **E**. A comminuted fracture in the proximal segment of the right clavicle (arrow). Significant soft tissue damages with a retrosternal hematoma (*) is presented. **F**. A displaced fracture of the manubrium. The cardiac displacement and significant subcutaneous emphysema here are caused by a right-sided tension pneumothorax identified in other views (arrow).

Hemostasis in patients with traumatic bleeding shock is the greatest challenge for traumatologists. A heparin-minimized VA-ECLS may be helpful for patients receiving a protracted damage-control procedure and developing CA later owing to prolonged hypoperfusion. Our case 3 was an interesting example in this category. He received an exploratory right thoracotomy for a massive hemothorax. After removing mediastinal blood clots, an unexpected hemorrhage from the right atrium through a pericardial laceration was noted. The patient then developed CA during cardiac manipulations to repair the lacerated right auricle and was unresponsive to direct cardiac massage, plus epinephrine injections. A peripheral VA-ECLS accompanied by aggressive blood transfusion restored the perfusion and allowed the patient to survive to hospital discharge. With a retrospective review of the preoperative CT, in addition to the previously noted right-side pneumothorax and hemopericardium, a tiny pneumopericardium was identified and considered a strong evidence of this rare phenomenon [11] (Figure 1A and B).

Patients with post-traumatic hypoxic CA in this study were characterized by high-grade thoracic injury. They showed a hypoxic CA due to traumatic asphyxia on the scene or a profound respiratory dysfunction in spite of mechanical ventilation (MV). Offering VA-ECLS to patients with post-traumatic hypoxic CA caused by a rare but often lethal injury is a dilemma for ECLS specialists [12], because an expensive but futile medical care may be done. The case 5, a victim of high-voltage electrocution, is a typical example. The VA-ECLS was performed to reverse the post-traumatic hypoxic CA developing soon after his arrival. The CT on ECLS showed no obvious internal hemorrhage but profound thermoelectric damages to the chest wall, lungs and intra-abdominal organs (Figure 1C and D). The VA-ECLS was terminated after 17 h due to persistent hypotension and metabolic acidosis.

It is also noteworthy that the ECLS team may occasionally need to switch VA-ECLS to VV-ECLS in patients with a myocardial recovery from post-traumatic hypoxic CA [13]. These patients develop severe post-traumatic ALI and fail to perform an acceptable gas exchange with native lungs during the weaning process from VA-ECLS. The post-traumatic ALI may be worsened continuously by superimposed infections related to prolonged intubation, non-healing wounds, and a compromised immunity accompanied with malnutrition and systemic inflammatory reactions on ECLS. In these cases, multiple organ failure due to overwhelming septic shock is always the cause of death on ECLS. Our case 8, a victim with flail chest due to combined fracture of the right clavicle and manubrium (Figure 1E and F), is a good example. Aside from the significant thoracic injury, he also had multiple long bone fractures and fixed with splints. The VA-ECLS was shifted to VV-ECLS after 96 h for purely pulmonary support. Unfortunately, a left-sided empyema and open osteomyelitis of the sternum/ left knee were found on VV-ECLS. Surgical debridements and fracture fixations were held over concerns of ECLS coagulopathy. The ECLS was terminated after 258 h due to an uncontrolled left-sided hemothorax associated with necrotizing pneumonitis. This experience made us reconsider and wonder if the post-traumatic ALI could be reversed with an early surgical debridement/reconstruction on ECLS.

The major limitation of the current study was a small and inhomogeneous database. Nevertheless, some valuable lessons could still be learned from a thorough case analysis. A clinical sensitivity of reversed injuries, a secure implantation of the device, and a proactive stance towards complications on ECLS should be important factors to reduce the risk of a futile VA-ECLS in trauma patients. Increasing the volume of practice is essential to build-up the institutional consensus and to develop a versatile program of post-traumatic VA-ECLS.

## Conclusion

Using VA-ECLS to treat post-traumatic shock/CA is challenging and requires multidisciplinary expertise.

## Competing interests

The authors declare they have no competing interests.

## Authors’ contributions

TYH, WTI, and LPJ contributed to the literature reviews, manuscript composition, and editing. WMY contributed to manuscript composition and editing and was responsible for the final product. LTC contributed to medical image reviews. All authors read and approved the final manuscript.
